# National Electronic Health Record Coverage in Pacific Island Countries and Territories: Environmental Scan

**DOI:** 10.2196/71212

**Published:** 2025-10-03

**Authors:** E Emily Porrello, Amy Peden, Michael Nunan, Rohina Joshi

**Affiliations:** 1 School of Population Health Faculty of Medicine & Health UNSW Sydney Sydney Australia; 2 Beyond Essential Systems Melbourne Australia; 3 Discipline of Public Health and Tropical Medicine College of Public Health, Medical and Veterinary Sciences James Cook University Townsville Australia; 4 Health Systems Science The George Institute for Global Health Sydney Australia; 5 Global Centre for Human Resources for Health Intelligence Walter Sisulu University Mthatha South Africa

**Keywords:** electronic health record, electronic medical record, health information system, digital health, eHealth, Pacific Islands, Cook Islands, Federated States of Micronesia, Fiji, Kiribati, Marshall Islands, Nauru, Niue, Palau, Papua New Guinea, Samoa, Solomon Islands, Tonga, Tuvalu, Vanuatu

## Abstract

**Background:**

Pacific Island countries and territories (PICTs) face unique challenges in delivering health care and sustaining digital health systems. These challenges include geographically dispersed populations and service delivery points, workforce shortages, and poor infrastructure. National electronic health records (EHRs) can strengthen health systems by facilitating continuity of care but are only available in 47% of countries worldwide. The status of national EHRs in PICTs has not been previously described in the published literature.

**Objective:**

This study aimed to map national EHR coverage in 14 PICTs of the World Health Organization (WHO) Western Pacific Region classified as Small Island Developing States (SIDS). This included the presence or absence of a national EHR; identification of EHR software used; coverage nationally and across primary, secondary, and tertiary facilities; presence or absence of supporting digital health or health information system strategies and policies; comparison of national EHR coverage in PICTs with national EHR coverage globally; and exploration of a relationship between EHR coverage and country income in PICTs.

**Methods:**

Given the absence of peer-reviewed literature on EHRs in PICTs, an environmental scan methodology was selected to review gray literature sources. We conducted a 3-stage environmental scan to systematically search publicly available websites across government, bilateral, multilateral, and philanthropic organizations for documents describing the status of national EHR implementations in the aforementioned 14 PICTs.

**Results:**

Of the 14 PICTs assessed, 12 countries (86%) have an EHR implemented at some level of the public health system, and 8 (57%) have a single national system implemented at more than one facility. Although this is higher than national EHR coverage rates globally (57/122, 47%), average coverage across the 12 PICTs using EHRs was only 39% (median 16%). We also identified a positive relationship between EHR coverage and country income status and generally medium to high EHR coverage across tertiary hospitals (19/41, 46%) and secondary care facilities (29/77, 38%) but low implementation at primary care facilities (61/4158, 1.5%). EHR coverage across all facilities in the 14 countries assessed was 2.5% (108/4267). EHR software used includes Tamanu (Nauru, Palau, Samoa, Kiribati, Fiji [Aspen Medical public-private partnership hospitals]), Medtech (Cook Islands, Niue), Vesalius (Tonga), PATIS Plus (Fiji), and custom systems.

**Conclusions:**

Our findings demonstrate, for the first time, that EHRs are being implemented in PICTs, including at scale in some settings. Despite high apparent coverage in some PICTs, the success of implementation and health worker usage remains unclear. Gray literature indicates that some EHRs currently available are failing or incapable of scaling nationally. To support sustainability of national EHRs in PICTs, governments should prioritize the implementation of fit-for-purpose, open-source, and scalable EHRs, and future studies should assess the success of EHR adoption and impact in the region.

## Introduction

National electronic health records (EHRs) can strengthen health systems by facilitating continuity of care across community, primary, secondary, and tertiary care settings and by providing data for clinical and policy decision-making [1-6]. Implementation and sustainability of EHRs at a national scale are, however, extremely complex even in well-resourced settings. The status of national EHR implementations worldwide has been tracked by the World Health Organization’s (WHO) Global Observatory for eHealth (GOeH) since 2005, with the most recent review in 2015 finding a national EHR was available in 47% of WHO Member States surveyed [7]. However, the results have extremely limited applicability to the WHO Western Pacific region (44% response rate) [7] and especially to Pacific Island countries and territories (PICTs), with Kiribati the sole responder among the region’s PICTs [8]. The more recent Global Digital Health Monitor was relaunched at the World Health Assembly in May 2023 and measures indicators aligned with the WHO Global Digital Health Strategy 2020-2025 [9]. It found that 75% of countries surveyed had a national digital health strategy complete or in progress [9], which is considered a stepping stone toward the implementation of a national EHR [7]. Like the GOeH survey, however, the results of the Global Digital Health Monitor have extremely limited applicability to PICTs, with only a single PICT (Papua New Guinea) represented among 67 participating countries.

PICTs face major health system barriers including geographically dispersed populations and service delivery points, workforce shortages, and poor infrastructure [5,10] and are underrepresented in our current understanding of national EHR progress globally. Health system digitization has been a consistent priority of PICT leaders over the last decade [11-19], but the vision of an integrated digital ecosystem has been difficult to achieve, with multiple fragmented solutions introduced in several settings [1-4,17,20-23]. Globally, the COVID-19 pandemic produced a rise in digital health infrastructure maturity in many countries between 2019 and 2023 [9] indicating a potential for national EHRs to have progressed among PICTs. This environmental scan aimed to map the current status of national EHRs in 14 PICTs of the WHO Western Pacific Region classified as Small Island Developing States (SIDS) [24,25] and compare findings with known metrics in the global context.

For each country, the research questions were:

1. Is a national EHR present?
2. What EHR software is used?
3. What is the coverage of EHRs nationally and across primary, secondary, and tertiary facilities?
4. Are supporting digital health or health information system (HIS) strategies and policies available?

At the regional level, the questions were:

1. How does national EHR coverage in PICTs compare with national EHR coverage globally?
2. Is there a relationship between EHR coverage and country income in PICTs?

## Methods

### Definitions

The definition of an EHR remains under debate in the literature [26]. We adopted the definition of an “EHR” and “national EHR” used by the WHO’s GOeH in its pivotal 2015 Global Survey on eHealth [7]:

Electronic health records (EHRs) are real-time, patient-centred records that provide immediate and secure information to authorized users. EHRs typically contain a patient’s medical history, diagnoses and treatment, medications, allergies, immunizations, as well as radiology images and laboratory results. A National Electronic Health Records system is most-often implemented under the responsibility of the national health authority and will typically make a patient’s medical history available to health professionals in health care institutions and provide linkages to related services such as pharmacies, laboratories, specialists, and emergency and medical imaging facilities.

Like the GOeH survey, we used the terms EHR and electronic medical record (EMR) interchangeably, though EHRs are generally understood to encompass a wider range of health information than EMRs, which simply digitize traditional paper medical records and are typically implemented only in clinical settings [7]. This method aimed to ensure we captured all possible relevant patient-level systems in use.

When disaggregating national EHR status by health facility type, we also used the general classifications applied in the GOeH survey: “Primary care facilities include clinics and health care centres; secondary care facilities include hospitals and emergency care; tertiary care facilities include specialized care, and referral from primary/secondary care” [7].

Finally, when this study describes the “status of national EHRs,” this refers to the implementation of an EHR at health facilities in the public health system.

### Study Design

Librarian-verified literature searches conducted in PubMed, EMBASE, CINAHL, Scopus, and the Cochrane Library (CENTRAL) in January 2024 and February 2024 found no results relevant to the implementation of a national EHR in a PICT setting. Related literature in PICTs instead focused on digital health maturity assessments, information and communications technology (ICT) infrastructure, digital health policy, and the role of future interventions in achieving universal health coverage [10,19,27,28], indicating that PICTs are in the nascent stages of their national EHR journeys. An environmental scan methodology was therefore used to systematically search publicly available websites for documents describing the status of national EHR implementations in PICTs. As there is currently no recognized reporting guideline for environmental scans [29], a recent peer-reviewed study using an environmental scan methodology in PICTs was used to develop the core methodology [30] and adapted to the digital health context.

### Search Strategy

#### Overall Design

A 3-stage search was conducted for the 14 PICTs of the WHO Western Pacific Region classified as SIDS: Cook Islands, Federated States of Micronesia, Fiji, Kiribati, Marshall Islands, Nauru, Niue, Palau, Papua New Guinea (PNG), Samoa, Solomon Islands, Tonga, Tuvalu, and Vanuatu [24,25]. The steps were a (1) purposive search of known government repositories; (2) systematic Google Advanced search; and (3) purposive search of bilateral, multilateral, and philanthropic development partner repositories.

#### Government Repositories

The first author compiled a list of health and ICT government departments relevant to each country’s governance structure. The 4 authors reviewed and agreed upon the final list of 29 government department websites for inclusion (Multimedia Appendix 1).

#### Google Advanced

The first author conducted a systematic Google Advanced search for each country between April 1, 2024, and May 18, 2024, limited by date range (January 1, 2015, to April 1, 2024), PDF file format, and country domain. The country domains were [.ck] for Cook Islands, [.fm] for Federated States of Micronesia, [.fj] for Fiji, [.ki] for Kiribati, [.mh] for Marshall Islands, [.nr] for Nauru, [.nu] for Niue, [.pw] for Palau, [.pg] for Papua New Guinea, [.ws] for Samoa, [.sb] for Solomon Islands, [.to] for Tonga, [.tv] for Tuvalu, and [.vu] for Vanuatu. This aimed to identify local documents missed, or published outside, the official government repositories. If the results stated that some entries were omitted due to similarities, the search was repeated with the omitted results included.

The final search query used was as follows: “electronic health record” OR “electronic medical record” OR “electronic patient record” OR “digital health record” OR “digital medical record” OR “digital patient record” OR “digital health” OR “eHealth” OR “health information system” site:[Country Domain] filetype:pdf.

For Vanuatu, the same terms were translated in French using Google Translate and searched again in Google Advanced: “dossier de santé électronique” OR “dossier médical électronique” OR “dossier patient électronique” OR “dossier de santé numérique” OR “dossier médical numérique” OR “dossier patient numérique” OR “santé numérique” OR “e-Santé” OR “système d'information de santé” site:.vu filetype:pdf. These terms were verified by a bilingual native French speaker with digital health expertise to ensure these search strings retained the same meaning in English and French.

The search strings purposefully included a wide range of terms encompassing EHRs, EMRs, digital health, and HIS more broadly. Given the lack of globally accepted terminology around EHRs and EMRs, this intended to ensure that all possible terms used by different stakeholders intending to refer to EHRs were captured.

#### Bilateral, Multilateral, and Philanthropic Development Partner Repositories

The 4 authors identified and agreed upon the following key bilateral, multilateral, and philanthropic development partners operating in health systems in the Western Pacific region: WHO; the Pacific Community (SPC); World Bank; Asian Development Bank; Institute for Health Metrics and Evaluation; Pacific Islands Forum Secretariat; Clinton Health Access Initiative (CHAI); Bloomberg Philanthropies; Bill & Melinda Gates Foundation; CDC Foundation; Vital Strategies; Plan International; PATH; and the governments of Australia, China, Japan, New Zealand, and the United States of America (the top government development donors in the Pacific region) [31]. The first author compiled a list of relevant websites for each development partner (Multimedia Appendix 2). We applied a snowball sampling methodology such that any potentially important additional development partners identified through the initial list were also searched. A total of 29 websites covering 18 development partner organizations were searched.

### Document Identification Inclusion and Exclusion Criteria

Where an advanced search function was available, we used each website’s search function to identify documents likely to contain information about EHRs (using the same search terms as the Google Advanced search and, where available, date and country or Pacific regional filters). Where a website did not have an advanced search function, a manual search of the tabs or sections on the home page was conducted to identify any sections with potential relevance to national EHRs. The approach varied depending on the website structure: A reports, resources, or publications section was typically identified and manually searched.

Documents were selected for screening if they were (1) related to health or ICT systems of 14 PICTs of the WHO Western Pacific Region classified as SIDS; (2) an annual report; development progress report, strategy or policy; digital health report, strategy or policy; document arising from a meeting of Pacific Health Ministers; or another document considered potentially likely to include information related to national EHR status by expert opinion; (3) published since January 1, 2015 (aligning with the year of the last WHO GOeH survey); (4) freely accessible at the time of the search; (5) written in English (or French for Vanuatu, where French is an official language); and (6) except for 2 interactive-only websites describing country digital health status, in PDF format (as these were considered more likely to contain official published information than other formats). The exclusion criteria for documents were (1) having an access fee, (2) in a language other than English (or French for Vanuatu), or (3) in a non-PDF format (excluding the 2 exceptions mentioned in the previous sentence). If an EHR was identified using these steps, we searched the EHR vendor’s web page (if available) for further information.

### Document Screening and Data Extraction and Synthesis

A hierarchical approach was used to review documents selected for screening. We first looked for national EHR system updates in regional documents from bilateral, multilateral, and philanthropic development partners (as these were likely to contain information relating to multiple countries). We then reviewed country-specific documents from government repositories; the Google Advanced search; and bilateral, multilateral, and philanthropic development partners. Eligible documents were screened by searching for key terms (digit, electronic, eHealth, e-Health, EHR, EMR, information sys) within each document using the Adobe Acrobat PDF search function.

Documents were included for analysis if they provided information on a relevant country’s EHR or digital health status more broadly. We extracted information related to EHR and selected digital health foundations metrics analyzed in the WHO GOeH survey 2015 [7] and expanded this to include further qualitative details where available. Descriptive data were extracted to complete a predefined Excel data collection table including availability of a national eHealth policy or strategy, HIS policy or strategy, national EHR system, percentage (%) coverage of the national EHR system (disaggregated by primary, secondary, and tertiary facilities), and the relationship of other digital health systems used with the EHR (see Multimedia Appendix 3 for the data collection table). We also extracted qualitative data relating to in-practice EHR usage, where these data were available in the documents reviewed (discussed in the country-level situational analyses in Multimedia Appendix 4).

Two authors (EEP and AP) independently conducted a pilot search for Samoa to test the document selection methodology and dual screened a random 10% subset of retrieved documents against the inclusion and exclusion criteria to check for accuracy in April 2024, with discrepancies settled by consensus. EEP single searched the remaining websites and single screened the remaining documents, a decision based on the high interrater agreement, time and resource constraints, and alignment with the PRISMA (Preferred Reporting Items for Systematic Reviews and Meta-Analyses) guidelines and other environmental scans [30,32,33]. EEP extracted data for all included documents using the predefined Excel data collection table, and AP independently extracted data for a random 10% subset of documents across all included countries as a further quality check.

A mixed methods approach was used for data synthesis. We first quantified national EHR metrics by country, with an accompanying narrative summary of the current situation. A comparative analysis between PICTs and against known global metrics was undertaken, and we synthesized current gaps in national EHRs across PICTs with recommendations.

### Data Verification

Senior health or ICT leaders in each country were contacted by email in October 2024 and November 2024 to validate the findings. Findings were validated by leaders from 7 of the 14 countries (50% response rate), while the remaining countries did not respond to the request within the indicated timeframe.

### Ethical Considerations

Human research ethics review was not required for this study, which involved a review of gray literature only and no human participants [34].

## Results

### Retrieved Documents

**Figure 1:**
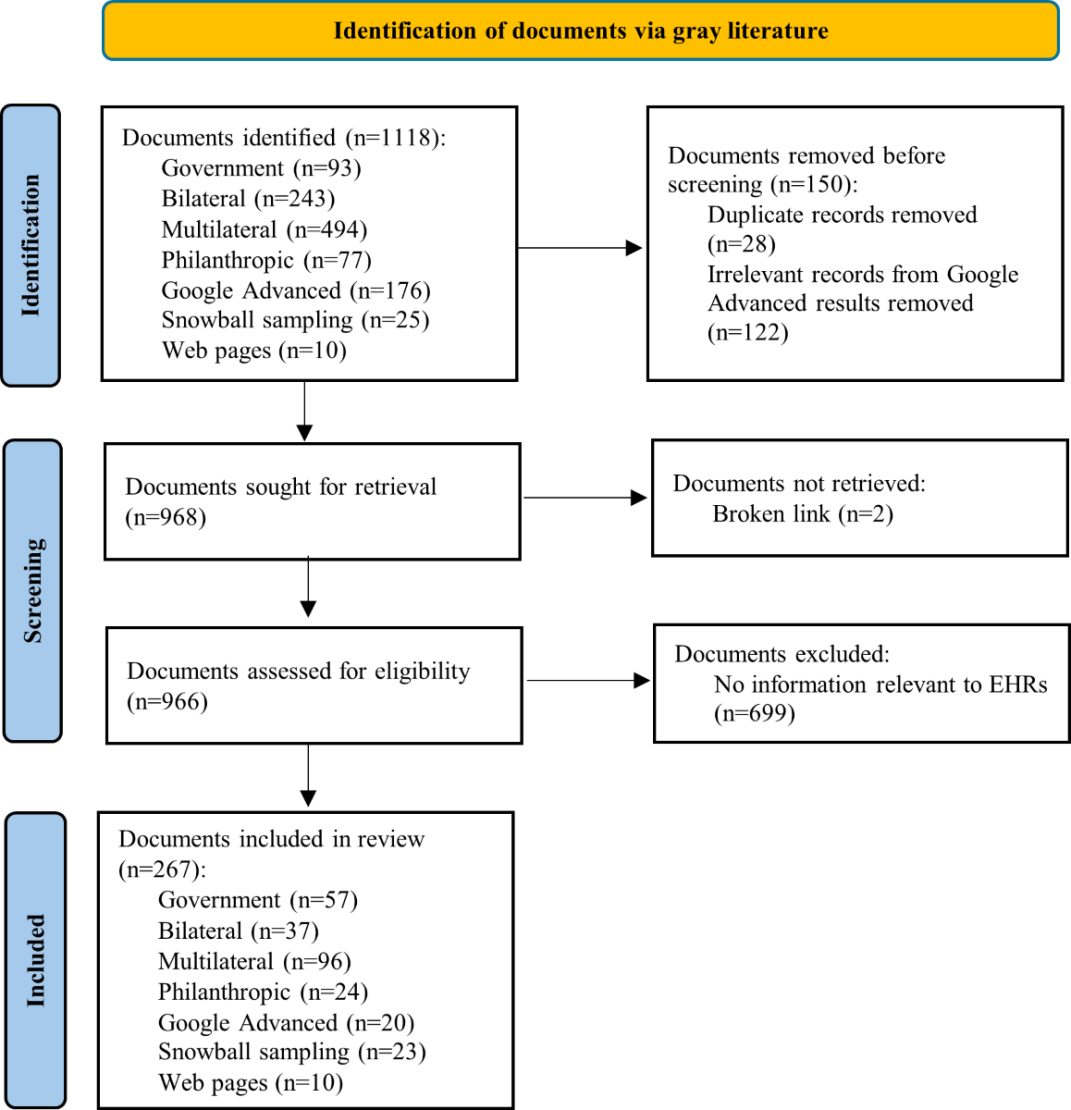
PRISMA (Preferred Reporting Items for Systematic Reviews and Meta-Analyses) flow diagram for identification of documents containing information about electronic health records (EHRs) in 14 Pacific Island countries and territories (PICTs) of the World Health Organization (WHO) Western Pacific Region classified as Small Island Developing States, via gray literature sources using an environmental scan methodology (April 1, 2024 to May 18, 2024).

A total of 1118 documents were identified, of which 966 were screened. Of these, 267 documents were included in the final review (see Figure 1 for the PRISMA flow diagram for identification of the literature).

**Figure 2:**
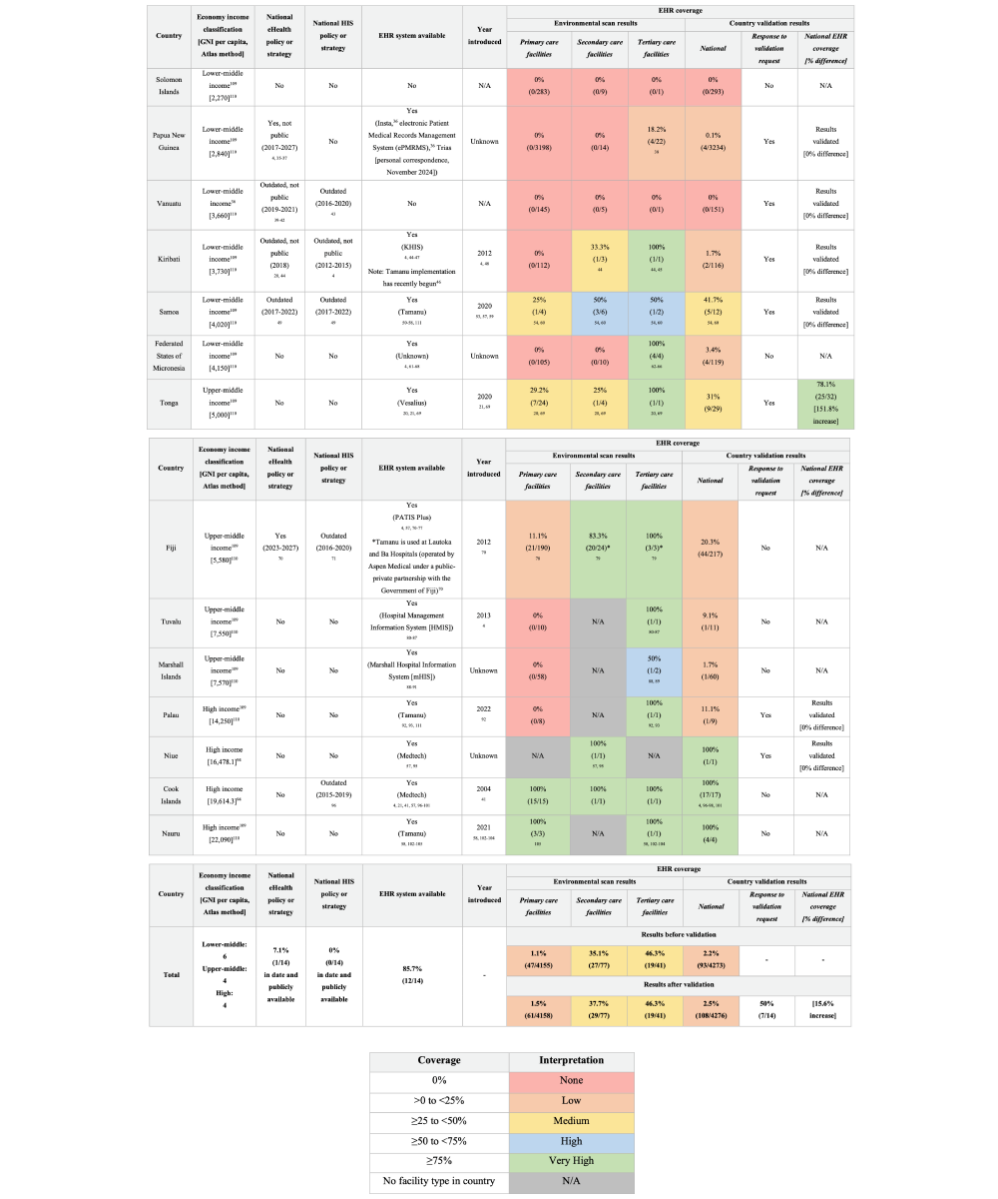
Electronic health record (EHR) coverage across primary, secondary, and tertiary care settings in 14 Pacific Island countries and territories (PICTs) of the World Health Organization (WHO) Western Pacific Region classified as Small Island Developing States, identified using an environmental scan methodology of gray literature sources (April 1, 2024, to May 18, 2024). For the percent difference in national EHR coverage for Tonga, see Multimedia Appendix 4. Superscript numbers indicate reference citations. ePMRMS: electronic patient medical records management system; GNI: gross national income; HIS: health information system; HMIS: hospital management information system; N/A: not applicable. A higher resolution version of the image is available in Multimedia Appendix 5 [4, 20, 21, 35-111].

The results of the environmental scan and validation step are summarized in Figure 2 [4, 20, 21, 35-111]. In the figure, the economy income classification was determined using World Bank Income Classifications 2025 Fiscal Year [109]. For countries not listed (Cook Islands and Niue), income status was determined using gross national income (GNI) per capita (Atlas method) values from the most recent data in the Asian Development Bank Key Indicators Database (Cook Islands: 2023; Niue: 2021) [94] then classified according to World Bank categories: For the current 2025 fiscal year, low-income economies are defined as those with a GNI per capita, calculated using the World Bank Atlas method, of US $1145 or less in 2023; lower middle-income economies are those with a GNI per capita between US $1146 and US $4515; upper middle-income economies are those with a GNI per capita between US $4516 and US $14,005; and high-income economies are those with a GNI per capita of more than US $14,005 [110].

### Availability of EHRs in PICTs

Of the 14 PICTs assessed, 12 countries (86%) have an EHR implemented at some level of the public health system. Eight countries (57%) have a single national system implemented at more than one facility (including Niue, which only has one health facility). This is higher than the global EHR coverage figure (57/122, 47%) from the most recent GOeH survey in 2015, indicating that PICTs may be “leapfrogging” other countries in their national EHR journeys. Notably, only a single PICT (Kiribati) was represented in the GOeH survey results [7], but EHRs in Cooks Islands, Fiji and Tuvalu were introduced prior to 2015. Four additional PICTs (Nauru, Palau, Samoa, Tonga) have introduced EHRs since 2020. EHRs currently used in the region are Tamanu (Nauru, Palau, Samoa, Kiribati, Fiji [at facilities operated by Aspen Medical under a public-private partnership with the Government of Fiji]), Medtech (Cook Islands, Niue), Vesalius (Tonga), and PATIS Plus (Fiji), as well as custom systems in other countries. Of the EHRs in use, only Tamanu is an approved Global Digital Good for Health, which are impactful, scalable, and adaptable open-source digital health tools suitable for low- and middle-income countries (LMIC) [111].

### National Coverage of EHRs in PICTs

Despite most PICTs using EHRs at some level of the public health system, average national coverage across the 14 countries assessed was 33% (median 10%). Of the 12 countries using EHRs, average national coverage was 39% (median 16%), though there was wide variation in EHR coverage between countries (range: 0.1%-100%). Fair comparison is challenging, as some countries achieved 100% coverage relatively easily due to having a small number of facilities (eg, Niue) compared with significantly larger countries with thousands of facilities (eg, PNG).

Our results indicate a positive relationship between EHR coverage and country income status for PICTs (Table 1). This mirrors a global trend identified in the GOeH survey, though that study was limited by low response rates for LMIC [7]. Within the lower-middle income countries assessed in our study, Samoa was a leader, with 42% (5/12) of facilities having a national EHR, compared with less than 5% coverage for all other PICTs in that category. In the upper-middle countries, a reverse trend was observed, with the least resourced countries outperforming the others and Tonga leading the group with 78% (25/32) coverage. All but one high-income country assessed had achieved 100% national EHR coverage, though this group includes some small countries with very few facilities (Niue and Nauru).

**Table 1:** National coverage of electronic health records (EHRs) across health facilities in 14 Pacific Island countries and territories (PICTs) of the World Health Organization (WHO) Western Pacific Region classified as Small Island Developing States (SIDS) identified using an environmental scan methodology of grey literature sources (April 1, 2024, to May 18, 2024), ordered by gross national income (GNI).

Country			Facilities, n		EHR coverage, n (%)
**Lower-middle income**					
	Solomon Islands	293		0 (0)	
	Papua New Guinea	3234		4 (0.1)	
	Vanuatu	151		0 (0)	
	Kiribati	116		2 (1.7)	
	Samoa	12		5 (41.7)	
	Federated States of Micronesia	119		4 (3.4)	
**Upper-middle income**					
	Tonga	32		25 (78.1)	
	Fiji	217		44 (20.3)	
	Tuvalu	11		1 (9.1)	
	Marshall Islands	60		1 (1.7)	
**High income**					
	Palau	9		1 (11.1)	
	Niue	1		1 (100)	
	Cook Islands	17		17 (100)	
	Nauru	4		4 (100)	

Of the 7 countries with validated data, only Tonga had significant differences in national EHR coverage compared with the initial findings of the environmental scan (78% vs 31%), due to their EHR rollout currently being underway (Figure 2). The remaining countries validated our findings, and some provided additional qualitative information (see Multimedia Appendix 4).

### EHR Coverage Across Primary, Secondary, and Tertiary Facilities in PICTs

**Figure 3:**
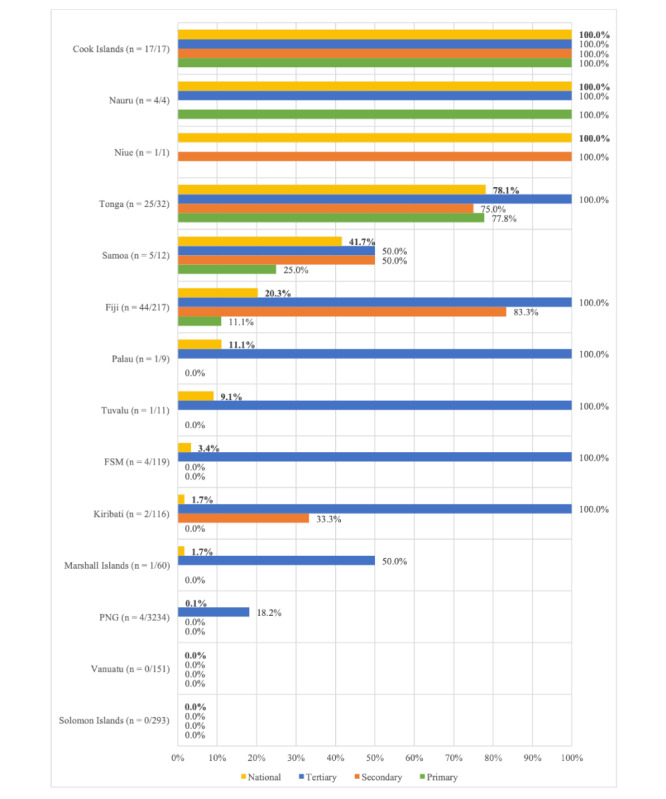
National electronic health record (EHR) coverage across primary, secondary, and tertiary health facilities in 14 Pacific Island countries and territories (PICTs) of the World Health Organization (WHO) Western Pacific Region classified as Small Island Developing States (SIDS), identified using an environmental scan methodology of gray literature sources (April 1, 2024, to May 18, 2024). FSM: Federated States of Micronesia; PNG: Papua New Guinea.

Within each PICT assessed, EHR coverage across tertiary care settings was generally very high, with full coverage in Cook Islands, Fiji, Federated States of Micronesia, Kiribati, Nauru, Palau, Tonga, and Tuvalu and 46% (19/41) of all tertiary facilities across the region using EHRs (Figure 3). In contrast, EHRs were only implemented in 1.5% (61/4158) of primary care facilities and limited to Cook Islands, Fiji, Nauru, Samoa, and Tonga. An EHR was implemented in only 2.5% (108/4276) of all primary, secondary, and tertiary health facilities in the region, with minimal EHR use in primary health facilities being the main contributor to low overall coverage. Even excluding PNG (which contributes a disproportionately high number of total health facilities compared with other countries assessed), an EHR was implemented in only 10% (104/1042) of all facilities.

### Availability of Digital Health and HIS Strategies and Policies in PICTs

Of the 14 PICTs assessed, only Fiji had an in-date national eHealth or HIS policy or strategy that was publicly available. Cook Islands, Kiribati, PNG, Samoa, and Vanuatu had relevant policies or strategies, but these were either no longer in date or not publicly available. Of the 4 countries that introduced an EHR since 2020 (Palau, Nauru, Samoa, Tonga), only 1 (Samoa) had a relevant, in-date policy or strategy during the time of EHR implementation.

## Discussion

### Principal Findings

Our findings demonstrate, for the first time, that EHRs are being implemented in PICTs (12/14, 86%), including at scale in some settings, with coverage generally higher in tertiary facilities than in primary care settings. These findings contrast with those of the GOeH survey, which found relatively uniform—and high (>80%)—coverage across primary, secondary, and tertiary settings in the 57 countries using a national EHR [7]. This difference is perhaps not surprising, however, given the low representation of LMIC in the GOeH survey and lack of digital health–supporting infrastructure, such as electricity and internet, in more remote locations where primary health facilities in PICTs (and other LMIC) are typically positioned. Interestingly, the GOeH survey found higher reported *usage* of EHRs in primary care facilities, with progressively lower usage in secondary and tertiary care facilities [7]. This would be an interesting focus for future research to explore in PICTs once implementation across primary care settings improves.

Despite high apparent EHR coverage in some PICTs, however, the success of implementation and in-practice usage by health workers remains unclear. Cook Islands, for example, theoretically has 100% national EHR coverage with Medtech, but usage at outer islands is poor due to internet connectivity and IT infrastructure issues [4,96-98,101]. Similarly, internet connectivity and data quality issues mean Fiji’s PATIS Plus system is now largely limited to the 3 major hospitals for accessing pathology results with less use for other clinical actions, despite a wider rollout having previously occurred [70,73]. Such examples highlight the need for fit-for-purpose EHRs that can operate in the geographically challenging settings of PICTs, from community outreach services to primary, secondary, and tertiary health facilities. There are technical requirements in achieving this, particularly for remote primary health facilities, which our results show make up most health services in the region but currently have very low EHR coverage. The EHR must be able to operate offline [5], be scalable, and be capable of interoperability with other software in the national digital health ecosystem. Although many complex factors affect EHR uptake, the gray literature indicates that some systems currently used in the region are failing or incapable of scaling to a national rollout because they lack one or more of these core technical features. To support sustainability of national EHRs in PICTs, governments should prioritize the implementation of fit-for-purpose, open-source, and scalable EHRs listed in Digital Square’s Global Digital Goods for Health Handbook [111].

Our study demonstrates that information about EHR status in PICTs is available, but it is scattered across government, bilateral, multilateral, and philanthropic organization documents and still not routinely captured despite multiple surveys and tools intending to track digital health status and maturity globally [9,106,107]. Only a single PICT (Kiribati) responded to the GOeH survey in 2015, and our study shows that, even when the survey was conducted, EHRs were available in at least three additional PICTs. This highlights a genuine lack of representation of PICTs in the GOeH survey and indicates a need to develop other methods of capturing these data effectively for PICTs. There is a need to better understand why PICTs were not well represented in the previous survey despite being invited to participate and why they remain underrepresented in more recently introduced tools tracking digital health metrics [9,106,107]. Alternative methodologies should be considered to improve representation of PICTs as this is a rapidly evolving space with at least 4 countries introducing EHRs since 2020 (likely incentivized by the COVID-19 pandemic) and national EHR rollouts currently underway in Samoa and Tonga. Our review also identified some confusion in the gray literature on the distinction between patient-level EHRs and aggregate health management information systems [108], such as District Health Information Software 2 (DHIS2) [111]. In certain settings (including Solomon Islands and Vanuatu), DHIS2 has some features that fulfill some requirements of an EHR (such as program registries). However, DHIS2 is not classified as an EHR [111] and therefore should not be considered equivalent to EHRs such as Tamanu, Medtech, and Vesalius.

A lack of up-to-date information about EHR status in PICTs and lack of clarity around the functions of different digital health systems in use are likely also producing inefficiencies. If development partners are not aware of national EHRs being implemented, they may introduce program-based EHRs limited to specific clinical use cases relevant to their practice or research. This practice inadvertently contributes to siloed health systems, rather than feeding into a single national system, and is problematic for long-term sustainability in the already resource-constrained health and ICT systems of the Pacific Islands. We emphasize the need for development partners supporting health system digitization in PICTs to improve coordination and visibility of their work to minimize the introduction of multiple project or program-based systems. To achieve this, however, easy access to up-to-date information about national EHRs (and other digital health systems) in use is required. This study provides a baseline for these data, but the challenge in maintaining it remains. To facilitate this, key organizations supporting digital health initiatives in the region (such as WHO, SPC, World Bank, Asian Development Bank, and the Australian Department of Foreign Affairs and Trade) should agree on a single method of tracking digital health metrics in PICTs, making similar overlapping tools obsolete and potentially mandating reporting into the selected system as part of funding arrangement requirements. Regional representative groups, such as the Pacific Health Information Network or the Asia eHealth Information Network (AeHIN), could be responsible for updating the status of digital health tools (including EHRs) in this system at their annual meeting with Pacific Island leaders working in this space.

This study provides the first holistic set of EHR data for PICTs, which could be used as a baseline for comparison with future studies or GOeH survey results (though PICTs had a very low response rate to previous surveys so may also have low response rates in the future). It provides a methodology for determining digital health status in PICTs and other LMIC, which were also underrepresented in the GOeH survey. In revealing the use of EHRs in PICTs, it creates a launching point for future research to evaluate the impact of these systems on health service delivery and health outcomes in PICTs. The question of “impact” of EHRs in LMIC more broadly is another gap in the literature, with the original GOeH survey and WHO Global Digital Health Strategy highlighting the lack of monitoring and evaluation of these tools. Our results also showed that in-date digital health policies or strategies were uncommon despite the presence of EHRs in the region. Future studies should explore whether the lack of a national digital health policy or strategy has impacted successful uptake and usage of EHRs in PICTs. Alternatively, if successfully implemented, this finding may challenge the idea that a fully formed digital health policy or strategy is a prerequisite to introduction of EHRs in PICT settings [74]. The EHR rollout may instead drive policy or strategy development, rather than the reverse.

### Limitations

There are several limitations to this environmental scan. Some relevant information may have been missed due to the search terms used; selection of government, bilateral, multilateral, and philanthropic organizations to review; document type restriction to PDF; unpublished data (gray literature authors were not contacted); human error during manual searches of websites that did not have advanced search functionality; and the use of a single searcher for the majority of document screening, which may introduce bias or oversight (a second author dual screened and extracted data for a random 10% subset of documents). It is also possible that the most up-to-date status of EHRs in PICTs is not yet reflected in official reports as it is a live space with EHR rollouts currently underway. We attempted to verify the findings with government stakeholders to minimize the risk of omissions (50% response rate), which showed there was no change to national EHR coverage for 6 of the 7 countries with verified data. In contrast, Tonga was significantly more advanced in their national EHR coverage than we identified in publicly available information, as their EHR rollout is currently in progress.

Our calculations of EHR coverage are based on each country’s total number of health facilities and classification of facility types as primary, secondary, or tertiary. It is still extremely challenging to find definitive, up-to-date lists of operational health facilities in PICTs, let alone classifications as primary, secondary, and tertiary. Some new (or recently re-operationalized) facilities may have been omitted, while some facilities no longer operational may have been included. Some facilities may have been incorrectly classified as primary, secondary, or tertiary. Potential errors in denominators could therefore affect the accuracy of calculated EHR coverage values, though any inaccuracies are likely to be minor. Again, we attempted to minimize this risk by verifying the findings with senior government stakeholders; however, in PICTs, it is often challenging, even at the government level, to verify the operational status of health facilities. The comparison of current EHR status in PICTs against global metrics from 2015 is also a limitation, as global metrics have also likely changed since then; however, this is the most up-to-date global information currently available for comparison and represents another gap in the literature. Finally, our study included 14 PICTs of the WHO Western Pacific Region classified as SIDS, and results may not be generalizable to other PICTs.

### Conclusions

This study demonstrates that several PICTs are more advanced in their EHR journeys than what has been formally reported. However, information is extremely challenging to access as it is scattered across gray literature in government and development partner resources. As EHR coverage continues to expand across the region, information must be more cohesively reported and accessible to enable PICT governments, practitioners, and academics to learn from the experiences of neighboring countries, which face unique challenges in health service delivery and digital health system maintenance. Although multiple EHRs have been implemented in PICTs, they have not been studied for adoption success or impact, with some indication in the gray literature of several systems failing. Global digital goods for health should be prioritized to promote affordability and sustainability of national EHRs in PICTs. Future research should prioritize investigation of EHR adoption success and impact in these settings and ensure information is shared to facilitate regional learning.

## Appendix

Multimedia Appendix 1 Government websites selected for searching.

Multimedia Appendix 2 Bilateral, multilateral, and philanthropic organization websites selected for searching.

Multimedia Appendix 3 Data collection table.

Multimedia Appendix 4 Country-level situational analysis.

Multimedia Appendix 5 Electronic health record (EHR) coverage across primary, secondary, and tertiary care settings in 14 Pacific Island countries and territories (PICTs) of the World Health Organization (WHO) Western Pacific Region classified as Small Island Developing States, identified using an environmental scan methodology of gray literature sources (April 1, 2024, to May 18, 2024).

Multimedia Appendix 6 PRISMA checklist.

## Abbreviations

AeHIN: Asia eHealth Information Network
CHAI: Clinton Health Access Initiative
DHIS2: District Health Information Software 2
EHR: electronic health record
EMR: electronic medical record
GNI: gross national income
GOeH: Global Observatory for eHealth
HIS: health information system
ICT: Information and communications technology
LMIC: low- and middle-income country
PICT: Pacific Island countries and territories
PNG: Papua New Guinea
PRISMA: Preferred Reporting Items for Systematic Reviews and Meta-Analyses
SIDS: Small Island Developing States
SPC: The Pacific Community
WHO: World Health Organization

## Data Availability

Data sets for this research are available in Multimedia Appendix 3.
