# Supplementary material for: National Electronic Health Record Coverage in Pacific Island Countries and Territories: Environmental Scan
Source: J Med Internet Res. 2025 Oct 3;27:e71212. doi: 10.2196/71212 (PMC12534756; doi:10.2196/71212)
Supplement: Multimedia Appendix 1 [file jmir_v27i1e71212_app1.pdf]

Multimedia Appendix 1: Government websites selected for searching

Country	Government Department/Ministry	Website
**Cook Islands**	Te Marae Ora – Cook Islands Ministry of Health	https://www.health.gov.ck/
	Office of the Prime Minister – Information Communication Technology	https://www.pmoffice.gov.ck/our-work/ict/
**Federated States of Micronesia**	FSM Department of Health and Social Affairs	https://hsa.gov.fm/
	Pohnpei State, Department of Health and Social Services	https://pohnpeistate.gov.fm/department-of-health-and-social-services-2/
	Yap State, Department of Health Services	https://www.yapstate.gov.fm/
	FSM Department of Transportation, Communication & Infrastructure	https://tci.gov.fm/index.html
**Fiji**	Ministry of Health and Medical Services	https://www.health.gov.fj/
	Ministry of ITC	* https://www.itc.gov.fj/
	digitalFIJI	https://www.fiji.gov.fj/digitalFIJI
**Kiribati**	Ministry of Health and Medical Services	* https://mhms.gov.ki/
	Ministry of Information, Communications & Transport	https://www.mict.gov.ki/
**Marshall Islands**	Ministry of Health and Human Services	https://rmihealth.org/
**Nauru**	Nauru RON Hospital	http://www.naurugov.nr/government/departments/department-of-health-and-medicinal-service.aspx
	Department of ICT	http://www.nauru.gov.nr/government/departments/department-of-telecommunications.aspx
**Niue**	Government of Niue	https://www.gov.nu/
**Palau**	Ministry of Health and Human Services	https://www.palauhealth.org/
	Palau National Government	https://www.palaugov.pw/
**Papua New Guinea**	Department of Health	https://www.health.gov.pg/
	Department of Information and Communications Technology	https://www.ict.gov.pg/
**Samoa**	Ministry of Health (MOH)	https://www.health.gov.ws/
	Ministry of Communications & Information Technology	https://mcit.gov.ws/
**Solomon Islands**	Ministry of Health and Medical Services	https://solomons.gov.sb/ministry-of-health-medical-services
	SIG ICT Services, Ministry of Finance & Treasury	https://solomons.gov.sb/ministry-of-finance-and-treasury/sig-ict-services/
**Tonga**	Ministry of Health (MoH)	http://www.health.gov.to/
	Digital Transformation Department, Department of Prime Minister's Office Tonga	https://digitaltransformation.gov.to/
	Information Technology and Communication Division, Ministry of Finance	https://finance.gov.to/ict
**Tuvalu**	Minister Of Health, Social Welfare And Gender Affairs	* https://www.gov.tv/minister-of-health-social-welfare-and-gender-affairs/
**Vanuatu**	Ministry of Health	https://moh.gov.vu/
	Office of the Government Chief Information Officer (OGCIO)	https://ogcio.gov.vu/
	**Total**	**29**

* At the time of searching, these websites were either not loading or the link to the 'Publications' (or equivalent) page was broken. We attempted to access these pages three separate times between May and September 2024.
