# Supplementary material for: National Electronic Health Record Coverage in Pacific Island Countries and Territories: Environmental Scan
Source: J Med Internet Res. 2025 Oct 3;27:e71212. doi: 10.2196/71212 (PMC12534756; doi:10.2196/71212)
Supplement: Multimedia Appendix 2 [file jmir_v27i1e71212_app2.pdf]

***Multimedia Appendix 2: Bilateral, multilateral, and philanthropic organisation websites selected for searching***

| Organisation | Website |
|---|---|
| **Bilateral** | |
| ***Australian Government*** | |
| Australian Department of Foreign Affairs & Trade (DFAT) | https://www.dfat.gov.au/ |
| Indo-Pacific Centre for Health Security (CHS) | https://indopacifichealthsecurity.dfat.gov.au/ |
| ***New Zealand Government*** | |
| NZ Ministry of Foreign Affairs & Trade (MFAT) | https://www.mfat.govt.nz/ |
| NZ Ministry of Health (MoH) | https://www.health.govt.nz/ |
| ***China Government*** | |
| China International Development Cooperation Agency (CIDCA) | http://en.cidca.gov.cn/ |
| Ministry of Foreign Affairs of the People's Republic of China | https://www.fmprc.gov.cn/mfa_eng/ |
| ***Japan Government*** | |
| Japan International Cooperation Agency (JICA) | https://www.jica.go.jp/english/ |
| Ministry of Foreign Affairs of Japan | https://www.mofa.go.jp/ |
| ***USA Government*** | |
| USAID | https://www.usaid.gov/ |
| US Department of State (DOS) | https://www.state.gov/ |
| **Multilateral** | |
| ***World Health Organization (WHO)*** | |
| WHO | https://www.who.int/ |
| WHO IRIS | https://iris.who.int/ |
| WHO-UNICEF Digital Health Centre of Excellence | https://www.digitalhealthcoe.org/ |
| WHO Digital Health Atlas | *https://digitalhealthatlas.org/en/-/ |
| Global Digital Health Monitor | https://monitor.digitalhealthmonitor.org/ |
| ***SPC*** | |
| SPC | https://www.spc.int/ |
| SPC Public Health Division | https://php.spc.int/ |
| SPC Statistics for Development Division | https://sdd.spc.int/ |
| ***World Bank*** | |
| World Bank | https://www.worldbank.org/en/home |
| ***ADB*** | |
| ADB | https://www.adb.org/ |
| ***IHME*** | |
| IHME | https://www.healthdata.org/ |
| ***Pacific Islands Forum Secretariat*** | |
| Pacific Islands Forum Secretariat | https://forumsec.org/ |
| **Philanthropic** | |
| ***Clinton Health Access Initiative (CHAI)*** | |
| CHAI | https://www.clintonhealthaccess.org/ |
| ***Bloomberg Philanthropies*** | |
| Bloomberg Philanthropies | https://www.bloomberg.org/ |
| ***Bill & Melinda Gates Foundation*** | |
| Bill & Melinda Gates Foundation | https://www.gatesfoundation.org/ |
| ***CDC Foundation*** | |
| CDC Foundation | https://www.cdcfoundation.org/ |
| ***Vital Strategies*** | |
| Vital Strategies | https://www.vitalstrategies.org/ |

| | |
|---|---|
| ***Plan International*** | |
| Plan International | https://plan-international.org/ |
| ***PATH*** | |
| PATH | https://www.path.org/ |
| **Total** | **29** |

* The Digital Health Atlas webpage became deactivated during the course of the study, and was later re-launched in the Implementome platform at https://gdhub.unige.ch/implementome/projects/source/dha
