# Supplementary material for: National Electronic Health Record Coverage in Pacific Island Countries and Territories: Environmental Scan
Source: J Med Internet Res. 2025 Oct 3;27:e71212. doi: 10.2196/71212 (PMC12534756; doi:10.2196/71212)
Supplement: Multimedia Appendix 4 [file jmir_v27i1e71212_app4.pdf]

Multimedia Appendix 4: Country-level situational analysis

Cook Islands

While all health facilities in Cook Islands have access to an EHR (Medtech), the extent of usage beyond Rarotonga Hospital is unclear with poor internet connectivity and IT infrastructure impacting use in the outer islands.[1-5] Despite its wide availability, the use of data for decision-making from Medtech also remains low.[3] There are challenges with data extraction and limited integration with other systems such as mSupply (inventory management) and systems for human resources information management, finance and public health surveillance.[3, 6]

A Request for EOI for Development of a Digital Health Strategy for Cook Islands was issued in May 2021.[6] However, it is unclear whether this eventuated as no eHealth policy or strategy is publicly available. There is no mention of health in the National Digital Strategy 2024-2030.[7]

A senior health leader in Cook Islands was contacted to validate the findings, but did not respond within the indicated timeframe.

Federated States of Micronesia

An EHR is available at the 4 major hospitals (Chuuk, Kosrae, Pohnpei, Yap),[8-12] but it is not named in any public documents retrieved during our search. It is unclear whether electronic patient records across the 4 hospitals are linked. The goal of a 2020 bid announcement issued by the FSM Department of Health & Social Affairs was to further develop the EHR to "centralize all patient activities in one central location so that wherever a patient in the FSM is seeking healthcare in any of the hospitals, provider (doctors and nurses) will have access to his/her medical record",[12] indicating that the sites may not have been connected previously. In addition, the extent of EHR use within the hospitals is unclear,[13] and lack of unique patient identification processes are an ongoing issue.[4, 10, 11, 14] Expansion of the EHR to lower-level facilities is not possible due to IT infrastructure issues including poor internet connectivity.[10, 11, 14]

A senior health leader in FSM was contacted to validate the findings, but did not respond within the indicated timeframe.

Fiji

Improving national EHR coverage has been listed as a key objective of the Ministry of Health and Medical Services (MHMS) since at least 2015 (the earliest government document reviewed in this study).[15] At the time, only 7 (13%) hospitals were reported to be using PATIS Plus.[15] While national EHR coverage has increased,[16, 17] PATIS Plus is not fully functional at existing sites and there are major data quality issues.[18, 19] For example, it contains significantly more National Health Numbers than the entire population of Fiji, indicating a multitude of duplicate patient records.[19] PATIS Plus usage is particularly poor at subdivisional hospitals and lower-level facilities, and now largely limited to the three major hospitals for accessing pathology results with less use for other clinical actions.[19] It is integrated with Fiji's laboratory information management system (StarLIMS)[4, 19-21] and births, deaths and marriages system at a subset of hospitals.[22, 23]

PATIS Plus requires a live connection to a central server[18, 19, 24] and uptake has been hindered by internet connectivity and infrastructure issues.[19] Given Fiji's most resourced health facilities (divisional and subdivisional hospitals) are among the existing sites, national expansion is likely not feasible due to even more constrained internet connectivity and IT infrastructure in remote locations. Replacing PATIS Plus has been a strategic goal of the MHMS since at least 2016,[25, 26] but this has not yet eventuated. Fiji's first Digital Health Strategy (released in 2023) describes PATIS Plus as a "patient administration system with limited EMR functionality", and reiterates a clear intention to replace it with a fit-for-purpose national EHR, starting by the end of 2027 with completion in 3-4 years.[19]

A senior health leader in Fiji was contacted to validate the findings, but did not respond within the indicated timeframe.

Kiribati

Kiribati Health Information System (KHIS) is a bespoke patient information system supported by the Japan International Cooperation Agency (JICA) and used at Tungaru Central Hospital, primarily in the medical records unit and medical ward, and Betio Hospital.[27-29] Access to and usage of KHIS are inconsistent, with a mix of electronic and paper-based records in use.[27-30] MHMS acknowledges that KHIS is not meeting the needs of clinicians, and is prioritising health system digitisation.[31-33] The bespoke nature of KHIS means expansion would require

significant investment for scale-up, and transition towards a digital public good has been recommended.[33] Kiribati MHMS has recently begun the implementation of Tamanu.[28]

There is no current eHealth Strategy in Kiribati, but the National ICT Policy 2019 describes a vision of a national EHR, working towards a "common medical records database" linking the major hospitals and clinics across the outer islands.[34] The National Health Strategic Plan 2020 to 2023 refers to a Digital Health Roadmap 2018,[27] but this is not publicly available.

A senior health leader in Kiribati validated the findings of this study (for Kiribati) [personal correspondence, November 2024].

Marshall Islands

Marshall Hospital Information System (mHIS) is a bespoke patient information system used at Majuro Hospital.[35-37] A consultancy was advertised in 2023 to develop a digital health strategy and undertake scoping for expansion of mHIS to outer islands.[38] The bespoke nature of mHIS means expansion would likely require significant investment for scale-up. It is cloud-based,[39] which would present challenges for implementation at remote facilities with poor connectivity. There is extremely limited public information available about mHIS and its use in Marshall Islands.

A senior health leader in Marshall Islands was contacted to validate the findings, but did not respond within the indicated timeframe.

Nauru

Implementation of Tamanu EHR at Republic of Nauru Hospital began in 2021, with the hospital rollout completed in 2023.[40-43] All hospital visits, radiology and lab requests, reports, and health ID cards are managed in Tamanu.[40] A national rollout of Tamanu to Naoero Public Health Centre and Nauru’s two operational Wellness Centres (Yaren and Anibare) was completed in 2024.[44]

Nauru does not have an eHealth/HIS strategy or policy.

A senior health leader in Nauru was contacted to validate the findings, but did not respond within the indicated timeframe.

Niue

Medtech has been implemented at Niue Foou Hospital (the country's only health facility), but current usage status is unclear.[22, 45] From the documents reviewed, it was unclear when Medtech was introduced, though its procurement is referenced as early as the Health Strategic Plan 2011-2021.[45]

According to the senior health leader who verified the results of this study for Niue, Medtech32 was first introduced at Niue Foou Hospital in 2006 and fully implemented in 2016 [personal correspondence, Nov 2024]. However, an outdated 1999 version of the system is still in use, with competing priorities hindering EHR progress in Niue [personal correspondence, Nov 2024]. Challenges include system underuse, lack of upgrades, ongoing costs, and aged infrastructure, and the question of whether to upgrade or overhaul Medtech in Niue is currently active [personal correspondence, Nov 2024].

Niue does not have an eHealth/HIS strategy or policy. The senior health leader who verified the study findings noted this was a priority of the Ministry of Health over the coming years [personal correspondence, Nov 2024].

Palau

Tamanu EHR was implemented at Belau National Hospital in 2022, with more than 59,000 patient encounters recorded in the first year.[46] EHR introduction began in 2020, as part of a holistic digital health project (Project Olangch), which entered its second phase in December 2024 [personal correspondence, Dec 2024]. Tamanu is also integrated with Palau's laboratory information management system SENAITE,[47] which was implemented in 2024 [personal correspondence, Dec 2024].

Palau does not have an eHealth/HIS strategy or policy.

A senior health leader in Palau validated the findings of this study (for Palau).

Papua New Guinea

PNG has an established electronic National Health Information System (eNHIS) which is used nationally for reporting aggregate health data.[4, 48-59] eNHIS is not considered an EHR, but

captures some patient-level data in program-specific modules (e.g. case-based registers for malaria and inpatients) [personal correspondence, Nov 2024]. Other disease-specific systems are also used sub-nationally, such as an electronic TB information management system (Bahmni) in Daru, Western Province.[60-63]

Publicly accessible documentation identified in this search noted that 'less than 5' facilities use EHRs or systems containing patient-level data.[55] Multiple systems are currently in use and are not interoperable nor considered scalable.[55] A system called Insta is used at Port Moresby General Hospital, and electronic Patient Medical Records Management System (ePMRMS) is used in Western Highlands Province.[55] Information about EHRs used at other facilities was not identified during our search; however, the senior health leader validating our findings for PNG noted that Trias is used at Kompiam Hospital and Kudjip Hospital [personal correspondence, Nov 2024]. They were not aware of any other sites using EHRs beyond the 4 mentioned above.

Increasing EHR coverage is a priority of the PNG National Department of Health,[64] and it is considering establishing a common EHR for all hospitals.[65] A goal of 100% coverage of provincial hospitals and Port Moresby General Hospital has been set for 2030.[55, 66] An assessment of all 22 government hospitals was undertaken in 2022,[67] but this is not publicly available.

A National eHealth Steering Committee has been established.[51, 64]

Several documents reviewed refer to a National eHealth policy/strategy,[4, 55, 56, 67, 68] but this is not publicly available. A senior health leader who verified the findings of this study for PNG noted that a Digital Health Strategy for 2025-2030 is currently awaiting endorsement from National Department of Health (NDoH) senior executive management [personal correspondence, Nov 2024].

Samoa

Tamanu EHR is implemented at Lalomanu District Hospital, Sa'anapu District Hospital, Sataua District Hospital, Safotu District Hospital, and Tupua Tamasese Meaole (TTM) Hospital, with a national rollout underway.[69, 70] Samoa's national EHR journey began in 2020[71, 72] with the implementation of Tamanu for COVID-19 immunisation tracking and NCD screening.[22, 41, 69, 72-75] Tamanu is replacing PATIS, which was previously only used at Samoa's two major

referral hospitals (TTM and MTII) and was no longer fit-for-purpose.[4, 76-79] At that time, most health data was manually collected through paper or Excel[78-81] and, according to the leader validating the findings for Samoa, this is still the case [personal correspondence, Nov 2024]. Tamanu implementation is currently in progress at TTM Hospital, with the system live at ENT Clinic, Ophthalmology Clinic, Orthopaedic Clinic, Orthotics and Prosthetics Clinic, Acute Primary Care Clinic (APCC), and the Expanded Program on Immunisation (EPI) [personal correspondence, Nov 2024].

Samoa's eHealth Policy and Strategy (2017-2022) describes a vision of a health information system that is linked across all community, clinical, population and public health facilities.[80] A consultancy to update this strategy was advertised in 2023,[71] but no revised document is publicly available.

Solomon Islands

The need for an EHR in Solomon Islands has been discussed by various stakeholders,[18, 82-85] but has not materialised. While a national EHR is not available, the patient-level module of DHIS2 ('Tracker') is implemented sub-nationally for immunisation delivery monitoring[86] and may fulfil some EHR functions for Solomon Islands. A bespoke SQL-based application called 'Patient Admissions, Discharges and Transfers (ADT) Summary' system has been implemented at the National Referral Hospital,[4] but does not appear to be used.

Solomon Islands does not have an eHealth/HIS strategy or policy.

A senior health leader in Solomon Islands was contacted to validate the findings, but did not respond within the indicated timeframe.

Tonga

Implementation of Tonga's National Health Information System (NHIS), which includes the Vesalius EMR,[87] began in 2020, and is used at Vaiola Hospital (2021), Niu'eiki Hospital (2022), and 7 health centres in Tongatapu (2022) according to publicly available documents identified in this study.[88-90] A national rollout is underway to outer island health facilities.[89] While the results of our review found the system had been implemented in 31.03% facilities (n = 9/29), Vesalius is now implemented at 25 of Tonga's 32 health facilities according to the senior health/ICT leader who verified our findings for Tonga. This includes 77.78% primary

care facilities (n = 21/27), 75% secondary care facilities (n = 3/4), and 100% tertiary care facilities (n = 1/1). This comprises all 16 facilities in Tongatapu, 4 of 5 facilities in Vava'u, 3 of 7 facilities in Ha'apai, and 'Eua's 2 facilities. Expansion of the NHIS to remaining facilities (including the single sites in Niuatoputapu and Niuafo'ou) is also planned. The NHIS replaced Tonga's previous HIS, a web-based Patient Administration System (WebPAS) customised for Vaiola Hospital (data was viewable by outer island hospitals but data entry was not possible).[91, 92]

Development of a digital health strategy was a key activity of ADB's multi-year e-Health project in Tonga that supported the Vesalius rollout.[91-93] According to the senior health/ICT leader who validated our findings for Tonga, the digital health strategy is in progress and targeted for completion by the end of 2024. A HIS and digital health roadmap was developed in 2022,[94] but is also not publicly available.

Tuvalu

Hospital Management Information System (HMIS) was implemented at Princess Margaret Hospital (PMH) in 2013[4] but it is not regularly used and not fit-for-purpose (designed for a tertiary hospital setting in Taiwan and still partially not translated).[95-102] HMIS does not integrate with Tuvalu's inventory management system mSupply.[98] World Bank planned to assess HMIS and provide a pathway for adapting or replacing it.[95, 96, 103] The strategy has not yet been completed, though it is still a target for December 2027.[102] There is no EHR outside PMH, and health records from clinics are recorded on paper or Excel.[4, 97]

Tuvalu does not have an eHealth/HIS strategy or policy.

A senior health leader in Tuvalu was contacted to validate the findings, but did not respond within the indicated timeframe.

Vanuatu

While a national EHR is not available, DHIS2 (VanPHIS2, VanHMIS) may fulfil some EHR functions for Vanuatu (such as program registries), though its function is primarily for aggregate data reporting rather than a longitudinal patient-level health record.[22, 104-107] A patient administration database is used at some hospitals; however, there is no link between health

facilities.[4] Multiple standalone digital health systems for individual programs are available,[106, 108] rather than all health data being managed holistically in a single national EHR.

A hospital EHR has been on Vanuatu's agenda for many years,[4, 108, 109] but has not yet progressed. Positively, there is a Digital Health Steering Committee[4, 110] and a Digital Health Strategy (though it is now outdated and not publicly accessible).[51, 109, 111, 112] WHO was engaged to develop a Digital Health Strategy for 2023-2025[107] but this is also not publicly available.

A senior health leader in Vanuatu validated the findings of this study (for Vanuatu).

References (Supplementary Files)

1. Te Marae Ora Ministry of Health Cook Islands. National Health Information Bulletin 2018. https://www.health.gov.ck/wp-content/uploads/2021/09/2018-bulletin-7-September2021-update.pdf Date accessed: May 20, 2024.
2. Te Marae Ora Ministry of Health Cook Islands. National Health Information Bulletin 2019-2020. https://www.health.gov.ck/wp-content/uploads/2022/04/TMO-health-bulltein-2019-2020-Final-20-April-2022.pdf Date accessed: May 20, 2024.
3. Te Marae Ora Ministry of Health Cook Islands. National Health Information Strategy Cook Islands: 2015-2019. https://www.health.gov.ck/wp-content/uploads/2020/06/National-Health-Information-Strategy-2015.pdf Date accessed: May 20, 2024.
4. World Health Organization. Regional action agenda on harnessing e-health for improved health service delivery in the Western Pacific. 2019. https://iris.who.int/bitstream/handle/10665/330700/9789290618959-eng.pdf?sequence=1&isAllowed=y Date accessed: April 14, 2024.
5. NTI and Brown University School fo Public Health Pandemic Center. Global Health Security Index Justification 2021: Cook Islands. 2021. https://ghsindex.org/wp-content/uploads/2021/12/Cook-Islands.pdf Date accessed: August 18, 2024.
6. World Health Organization. Request for EOI: Development of a Digital Health Strategy for Te Marae Ora Ministry of Health Cook Islands. 2021. https://www.ungm.org/Public/Notice/129594 Date accessed: May 25, 2024.
7. Cook Islands Office of the Prime Minister. Cook Islands National Digital Strategy 2024-2030. https://www.pmoffice.gov.ck/wp-content/uploads/2024/02/CI-National-Digital-Strategy-v10.pdf Date accessed: May 20, 2024.
8. FSM National Government, Department of Health and Social Affairs. Request for Proposals: Electronic Health Record Server Hardware Purchase and Installation Project. 2020. https://gov.fm/files/EHR_Servers_Request_for_Proposals_Final.pdf Date accessed: May 18, 2024.
9. Department of Health and Social Services Pohnpei State Government. DOHSS - Annual Report 2021. 2021. https://pohnpeistate.gov.fm/wp-content/uploads/2022/03/DOHSS-Annual-Report-2021.pdf Date accessed: May 20, 2024.
10. Wulf P, Sampson S. Digital Federated States of Micronesia Project Environmental and Social Management Plan. 2019. https://tci.gov.fm/documents/communications/digitalfsm/digital-fsm-esmp-final-2019.pdf Date accessed: May 20, 2024.
11. Sampson, S. Digital FSM Stakeholder Engagement Plan (Full Draft). 2019. https://tci.gov.fm/documents/communications/digitalfsm/digitalfsm-stakeholder-engagement-plan-no-track.pdf Date accessed: May 20, 2024.
12. FSM National Government, Department of Health and Social Affairs. Bid Announcement: Electronic Health Records Project. 2020. https://gov.fm/files/Announcements/Electronic_Health_Records_Project_BID.pdf Date accessed: May 18, 2024.
13. NTI and Brown University School fo Public Health Pandemic Center. Global Health Security Index Justification 2021: Federated States of Micronesia. 2021. https://ghsindex.org/wp-content/uploads/2021/12/Micronesia-Federated-States-of.pdf Date accessed: August 18, 2024.
14. World Bank. Project Appraisal Document: Digital Federated States of Micronesia Project. Report No: PAD3428. 2020. https://documents1.worldbank.org/curated/en/432601585596558171/pdf/Federated-

States-of-Micronesia-Digital-Federated-States-of-Micronesia-Project.pdf Date accessed: May 26, 2024.
15. Fiji Ministry of Health and Medical Services. Annual Corporate Plan 2016/17. https://www.health.gov.fj/wp-content/uploads/2023/12/Annual-Corporate-Plan-2016-2017.pdf Date accessed: May 20, 2024.
16. Fiji Ministry of Health and Medical Services. Annual Report 2019-20. https://www.health.gov.fj/wp-content/uploads/2023/12/Annual-Report-2019-2020.pdf Date accessed: May 20, 2024.
17. Fiji Ministry of Health and Medical Services. Annual Report 2020-2021. https://www.health.gov.fj/wp-content/uploads/2023/12/Annual-Report-2020-2021.pdf Date accessed: May 20, 2024.
18. Australian Department of Foreign Affairs and Trade, Office of Development Effectiveness. Strengthening Pacific Health Systems: Evaluating Ten Years of Australia's Support. 2019. https://www.dfat.gov.au/sites/default/files/pacific-health-evaluation.pdf Date accessed: April 6, 2024.
19. Fiji Ministry of Health and Medical Services. Fiji Digital Health Strategy 2023-2027. 2023. https://www.yumpu.com/en/document/view/68425187/fiji-digital-health-strategy-2023-2027-final/7 Date accessed: May 20, 2024.
20. Fiji Ministry of Health and Medical Services. Annual Report 2015. 2016. https://www.health.gov.fj/wp-content/uploads/2023/12/Annual-Report-2015-2016.pdf Date accessed: May 20, 2024.
21. UNICEF. Mapping of Digital Health Tools and Technologies: Fiji Country Brief. 2021. https://drive.google.com/drive/folders/1wsTqQKU4z0yFG11zGavCNN6BBatry8Gt?usp=drive_link Date accessed: August 17, 2024.
22. Pacific Community. Interoperability and data sharing between civil registration, health information, statistics and associated systems. 2024. https://spccfpstore1.blob.core.windows.net/digitallibrary-docs/files/41/410586426b1644bbfa27e6a7f930905d.pdf?sv=2015-12-11&sr=b&sig=hIleiTP8nHlu0rPEgGKpTB5CDxhdA3rXKIeIAXz%2FZ3c%3D&se=2025-05-30T23%3A42%3A45Z&sp=r&rscc=public%2C%20max-age%3D864000%2C%20max-stale%3D86400&rsct=application%2Fpdf&rscd=inline%3B%20filename%3D%22Interoperability_and_Data_Sharing_Report_2024.pdf%22 Date accessed: April 14, 2024.
23. Fiji Ministry of Health and Medical Services. Annual Report 2018-19. https://www.health.gov.fj/wp-content/uploads/2023/12/Annual-Report-2018-2019.pdf Date accessed: May 20, 2024.
24. World Health Organization. Universal Health Coverage: Moving Towards Better Health, Action Framework for the Western Pacific Region. 2016. https://iris.who.int/bitstream/handle/10665/246420/9789290617563_eng.pdf?sequence=1&isAllowed=y Date accessed: April 14, 2024.
25. Fiji Ministry of Health and Medical Services. Strategic Plan 2020-2025. https://www.health.gov.fj/wp-content/uploads/2020/05/Strategic-Plan-2020-2025-1.pdf Date accessed: May 20, 2024.
26. Fiji Ministry of Health and Medical Services. HIS-CIS Strategy 2016-2020. https://www.health.gov.fj/wp-content/uploads/2018/03/HIS-CIS-Strategy-2016-2020.pdf Date accessed: May 20, 2024.
27. Government of Kiribati, Ministry of Health and Medical Services. National Health Strategic Plan 2020 - 2023. https://extranet.who.int/fctcapps/sites/default/files/2024-04/391_KIR%20MHMS%20strategic%20plan%20FINAL%20%2814Oct2022%29.pdf Date accessed: August 18, 2024.

28. World Bank. Project Appraisal Document: Kiribati Health Systems Strengthening Project (P176306). Report No: PAD4749. 2022. https://documents1.worldbank.org/curated/en/643411649085425281/pdf/Kiribati-Health-Systems-Strengthening-Project.pdf Date accessed: August 12, 2024.
29. World Bank and World Health Organization. Kiribati Health Sector Support Program Aide Memoire: Joint World Bank/WHO Health Team Visit 23 September to 1 October 2015. https://www.mfed.gov.ki/sites/default/files/Kiribati_Joint%20Donor%20Health%20Mission%20Aide%20Memoire%20%2012%20Oct.pdf Date accessed: May 18, 2024.
30. World Health Organization. Meeting on Health Information System Strengthening in Pacific Island Countries in Suva, Fiji, 27-29 June 2018: Meeting Report. 2018. https://iris.who.int/bitstream/handle/10665/275485/RS-2018-GE-42-FJI-eng.pdf?sequence=1&isAllowed=y Date accessed: April 14, 2024.
31. World Bank. Concept Project Information Document (PID) - Kiribati Health Systems Strengthening Project (P176306). Report No: PIDC31523. 2021. https://documents1.worldbank.org/curated/en/199051627666256884/pdf/Concept-Project-Information-Document-PID-Kiribati-Health-Systems-Strengthening-Project-P176306.pdf Date accessed: August 12, 2024.
32. World Bank. Implementation Status & Results Report - Kiribati Health Systems Strengthening Project (P176306). 2023. https://documents1.worldbank.org/curated/en/099072003212329457/pdf/P176306015269a00e0b06507e24497159b9.pdf Date accessed: August 12, 2024.
33. UNICEF. Mapping of Digital Health Tools and Technologies: Kiribati Country Brief. 2021. https://a78da35e-056b-4420-8e4f-41e3283327e3.usrfiles.com/ugd/b3c01f_59abc8ef72b44cdeb00a4f7879c0e0a5.pdf Date accessed: August 17, 2024.
34. Government of Kiribati. Kiribati National ICT Policy 2019. https://www.mict.gov.ki/sites/default/files/National%20ICT%20Policy.pdf Date accessed: May 20, 2024.
35. Republic of Marshall Islands Ministry of Health & Human Services. Quad Report FY 2018, FY 2019, FY 2020, FY 2021. https://rmihealth.org/component/dpattachments/?view=attachment&tmpl=component&id=15 Date accessed: May 20, 2024.
36. World Health Organization. Joint External Evaluation of IHR Core Capacities of the Republic of the Marshall Islands. Mission report: 23-26 September 2019. 2020. https://iris.who.int/bitstream/handle/10665/333361/9789240008168-eng.pdf?sequence=1 Date accessed: August 12, 2024.
37. Republic of Marshall Islands Minsitry of Health & Human Services. Advmeds Marshall Hospital Information System (mHIS). https://mail.rmihealth.org/about-mohhs/our-work/organization Date accessed: August 12, 2024.
38. World Health Organization. Digital Health Strategy Consultant (advertisement). 2023. https://untalent.org/jobs/digital-health-strategy-consultant Date accessed: August 12, 2024.
39. AdvMeds. mHIS. 2024. https://advmeds.com/hospital-information-system/ Date accessed: October 20, 2024.
40. Australian Department of Foreign Affairs and Trade. 2022-23 Nauru Development Program Progress Report. https://www.dfat.gov.au/sites/default/files/2022-23-nauru-development-program-progress-report.pdf Date accessed: 24 August, 2024.
41. PATH. Digital Square Global Goods Guidebook, Third Edition. 2022. https://static1.squarespace.com/static/59bc3457ccc5c5890fe7cacd/t/62a46059642f3b5127550bfe/1654939756182/Global+Goods+Guidebook+V3-Update-10-June-2022.pdf Date accessed: April 21, 2024.

42. Australian Department of Foreign Affairs and Trade. 2020-21 Nauru Development Program Progress Report. https://www.dfat.gov.au/sites/default/files/2020-21-nauru-development-program-progress-report.pdf Date accessed: August 24, 2024.
43. Australian Department of Foreign Affairs and Trade. 2021-22 Nauru Development Program Progress Report. https://www.dfat.gov.au/sites/default/files/2021-22-nauru-development-program-progress-report.pdf Date accessed: August 24, 2024.
44. Beyond Essential Systems. All smiles as Nauru completes world first EMR achievement. March 25, 2024. https://www.bes.au/all-smiles-as-nauru-completes-world-first-emr-achievement/ Date accessed: August 18, 2024.
45. NTI and Brown University School fo Public Health Pandemic Center. Global Health Security Index Justification 2021: Niue. 2021. https://ghsindex.org/country/niue/ Date accessed: August 18, 2024.
46. Beyond Essential Systems. Case Study: One year anniversary of Project Olangch in Palau. November 17, 2023. https://www.bes.au/case-study-one-year-anniversary-of-project-olangch-in-palau/ Date accessed: August 18, 2024.
47. Beyond Essential Systems. Tamanu integration with SENAITE goes live in Palau using FHIR. June 25, 2024. https://www.bes.au/tamanu-integration-with-senaite-goes-live-using-fhir/ Date accessed: August 18, 2024.
48. Indo-Pacific Centre for Health Security. State of the Region 2019: Health Security in the Indo-Pacific. 2019. https://indopacifichealthsecurity.dfat.gov.au/sites/default/files/2022-07/Indo-Pacific%20Health%20Security%20-%20State%20of%20the%20Region%202019.pdf Date accessed: April 8, 2024.
49. Australian Department of Foreign Affairs and Trade. Annual Report 2022-23. 2023. https://www.dfat.gov.au/sites/default/files/dfat-annual-report-2022-23.pdf Date accessed: April 8, 2024.
50. Pacific Community. Civil registration and vital statistics systems in the Pacific: overview of the organisation, status of systems, and levels of completeness of birth and death registration by country. 2023. https://sdd.spc.int/digital_library/civil-registration-and-vital-statistics-systems-pacific-overview-organisation Date accessed: April 14, 2024.
51. Asian Development Bank. Digital Health Implementation Guide for the Pacific; 2021. https://www.adb.org/sites/default/files/publication/677181/digital-health-implementation-guide.pdf Date accessed: April 21, 2024.
52. Asian Development Bank. Pacific Economic Monitor – July 2019. 2019. https://www.adb.org/sites/default/files/publication/514206/pem-july-2019.pdf Date accessed: April 21, 2024.
53. World Health Organization. Report of the Regional Director: The work of WHO in the Western Pacific Region, 1 July 2022 - 30 June 2023. 2023. https://iris.who.int/bitstream/handle/10665/372692/WPR-RC074-02-RDrep-2023-en.pdf?sequence=1 Date accessed: April 14, 2024.
54. Papua New Guinea National Department of Health. Data Management Competency Profiles for Provincial Health Information Officers and Medical Records Officers. 2023. https://www.health.gov.pg/pdf/CPP_2023.pdf Date accessed: May 20, 2024.
55. Papua New Guinea National Department of Health. Monitoring and Evaluation Strategic Plan for the National Health Plan (2021-2030). 2023. https://www.health.gov.pg/pdf/MELspF_2023.pdf Date accessed: May 20, 2024.
56. World Health Organization. SCORE for Health Data Technical Package: Papua New Guinea. 2022. https://cdn.who.int/media/docs/default-source/documents/ddi/score/country-profiles/who_score_png_en.pdf?sfvrsn=2c6d0b61_1 Date accessed: May 4, 2024.

57. NTI and Brown University School fo Public Health Pandemic Center. Global Health Security Index Justification 2021: Papua New Guinea. 2021. https://ghsindex.org/wp-content/uploads/2021/12/Papua-New-Guinea.pdf Date accessed: August 18, 2024.
58. UNICEF. Mapping of Digital Health Tools and Technologies: Papua New Guinea Country Brief. 2021. https://drive.google.com/drive/folders/1195Zp9lXgatlTMKzRA3Fqd3kDLhfgiWM Date accessed: August 17, 2024.
59. Asia Pacific Observatory on Health Systems and Policies. Independent State of Papua New Guinea Health System Review. 9(1); 2019. https://iris.who.int/bitstream/handle/10665/280088/9789290226741-eng.pdf?sequence=5&isAllowed=y Date accessed: August 31, 2024.
60. Australian Department of Foreign Affairs and Trade. 2022-23 Papua New Guinea Development Program Progress Reporting. https://www.dfat.gov.au/sites/default/files/2022-23-papua-new-guinea-development-program-progress-report.pdf Date accessed: August 24, 2024.
61. Rosewell A, Shearman P, Ramamurthy S, Akers R. Transforming the health information system using mobile and geographic information technologies, Papua New Guinea. Bull World Health Organ. 2021 May 1;99(5):381-387A. https://iris.who.int/bitstream/handle/10665/341214/PMC8061671.pdf?sequence=1&isAllowed=y Date accessed: August 31, 2024.
62. Pank NP, Aung A, Kama G, Murray A, Huang KL, Greig J, et al. Continuous quality improvement in a community-wide TB screening and prevention programme in Papua New Guinea. Public Health Action. 2024 Sep 1;14(3):97-104. doi: 10.5588/pha.24.0013.
63. Morris L, Hiasihri S, Chan G, Honjepari A, Tugo O, Taune M, et al. The emergency response to multidrug-resistant tuberculosis in Daru, Western Province, Papua New Guinea, 2014-2017. Public Health Action. 2019 Sep 21;9(Suppl 1):S4-S11. doi: 10.5588/pha.18.0074.
64. Government of Papua New Guinea. National health plan 2021-2030, volume 2A: Situation analysis, 2011-2020. 2021. https://hivpreventioncoalition.unaids.org/en/resources/papua-new-guinea-national-health-plan-2021-2030-volume-2a-situation-analysis-2011-2020 Date accessed: May 20, 2024.
65. Government of Papua New Guinea. National Health Plan 2021-2030. Volume 1: Policies and Strategies. 2021. https://hivpreventioncoalition.unaids.org/en/resources/papua-new-guinea-national-health-plan-2021-2030-volume-1-policies-and-strategies Date accessed: May 20, 2024.
66. Papua New Guinea National Department of Health. Indicator Compendium: Monitoring and Evaluation Framework for the National Health Plan 2021-2030. https://www.health.gov.pg/pdf/SPddf_2023.pdf Date accessed: May 20, 2024.
67. Pacific Community. PHIN Information Document: 7.3 PNG's experience in digital health preparation for digital health maturity assessment. 2023. https://php.spc.int/sites/default/files/p-related-files/2023-03/2023%20PHIN%20Agenda%2031%20ID%207.3%20PNG%20experience%20in%20digital%20health%20preparation%20for%20digital%20health%20maturity%20assessment%2028%20April%202023.pdf Date accessed: August 29, 2024.
68. Global Digital Health Monitor. Papua New Guinea (updated May 2023). https://monitor.digitalhealthmonitor.org/country_profile/PNG Date accessed: August 24, 2024.
69. Government of Samoa, Minsitry of Health. Annual Report 2020-2021 Financial Year. 2022. https://www.health.gov.ws/wp-content/uploads/2023/01/MOH-AR-20-21_ENGLISH-VERSION.pdf Date accessed: April 1, 2024.

70. Samoa Observer. Rural hospitals come online. November 26, 2023. https://www.samoaobserver.ws/category/samoa/106872 Date accessed: April 21, 2024.
71. Government of Samoa, Ministry of Health. Terms of Reference: Technical Assistance to Review and Develop the Digital Health Policy and Strategy 2022/23 - 2027. 2023. https://www.health.gov.ws/wp-content/uploads/2023/05/Apr2023_ToR-eHealth-Policy-and-Strategy-Full-Review-Development.pdf Date accessed: April 1, 2024.
72. Australian Department of Foreign Affairs and Trade. 2020-21 Samoa Development Program Progress Report. https://www.dfat.gov.au/sites/default/files/2020-21-samoa-development-program-progress-report.pdf Date accessed: April 6, 2024.
73. Australian Department of Foreign Affairs and Trade. 2022-23 Samoa Development Program Progress Reporting. https://www.dfat.gov.au/sites/default/files/2022-23-samoa-development-program-progress-report.pdf Date accessed: April 6, 2024.
74. Australian Department of Foreign Affairs and Trade. 2021-22 Samoa Development Program Progress Report. https://www.dfat.gov.au/sites/default/files/2021-22-samoa-development-program-progress-report.pdf Date accessed: April 6, 2024.
75. Government of Samoa, Ministry of Communications and Information Technology. Information and Communication Technology Sector Plan 2022/23-2026/27. 2022. https://mcit.gov.ws/wp-content/uploads/2023/06/ICTSP-2022-2027_English-Version_Signed.pdf Date accessed: April 6, 2024.
76. Government of Samoa, Ministry of Health. Annual Report Financial Year 2018/2019. https://www.health.gov.ws/wp-content/uploads/2023/01/MOH-AR-18-19_ENGLISH-VERSION.pdf Date accessed: April 1, 2024.
77. Government of Samoa, Minsitry of Health. Samoa Health Sector Plan 2008-2018: Full Review Report. https://www.health.gov.ws/wp-content/uploads/2023/02/HSP-2008-2018-Full-Review-Report.pdf Date accessed: April 1, 2024.
78. United Nations Development Programme and Samoa Bureau of Statistics. Strengthening the Birth Registration System in Samoa: Intelligence Report. 2022. https://sbs.gov.ws/documents/crvs/2023/Intelligence%20Report%20on%20Birth%20Registration%20System%20in%20Samoa.pdf Date accessed: April 1, 2024.
79. World Bank. Concept Stage Program Information Document (PID) - Samoa Health System Strengthening Program - (P164382). Report No: PIDISDSC24053. 2018. https://documents1.worldbank.org/curated/en/691981524060200384/pdf/Concept-Stage-Program-Information-Document-PID-Samoa-Health-System-Strengthening-Program-P164382.pdf.
80. Government of Samoa, Ministry of Health. Samoa eHealth Policy and Strategy 2017-2022. 2017. https://www.health.gov.ws/wp-content/uploads/2023/02/Samoa-eHealth-Policy-Strategy-2017-2022.pdf Date accessed: April 1, 2024.
81. World Bank. Samoa Health System Strengthening Program for Results Project. Report No: PAD3018. https://documents1.worldbank.org/curated/en/465671575860741445/pdf/Samoa-Health-System-Strengthening-Program-for-Results-Project.pdf Date accessed: April 21, 2024.
82. World Health Organization. WHO Country Cooperation Strategy 2018-2022: Pacific Island Countries and Areas. 2018. https://iris.who.int/bitstream/handle/10665/272806/WPRO-2017-DPM-027-pic-eng.pdf?sequence=1 Date accessed: January 29, 2024.
83. UNICEF. UNICEF's Digital Health & Information System Annual Report 2022. https://a78da35e-056b-4420-8e4f-41e3283327e3.usrfiles.com/ugd/55ae33_46767663b4dd4b0ebf2f6fe9d7e1793d.pdf Date accessed: April 14, 2024.

84. Solomon Islands Ministry of Health & Medical Services. Annual Report 2017. https://solomons.gov.sb/wp-content/uploads/2020/02/MHMS-2017-Annual-report.pdf Date accessed: May 20, 2024.
85. Solomon Islands Ministry of Health & Medical Services. National Health Strategic Plan 2016-2020. https://solomons.gov.sb/wp-content/uploads/2020/01/SI-NHSP2016-2020-50pp-FINAL-Optimise.pdf Date accessed: May 20, 2024.
86. UNICEF. Mapping of Digital Health Tools and Technologies: Solomon Islands Country Brief. 2021. https://drive.google.com/drive/folders/1Rv50gHQrSE3otGONQ5vlloQNfBvjuJj8 Date accessed: August 17, 2024.
87. Nova Group. Nova secures nationawide project to implement Vesalius in Tonga. March 2020. https://www.nova-hub.com/novanews/nova-secures-nationwide-project-to-implement-vesalius-in-tonga/ Date accessed: August 18, 2024.
88. Pacific Community. PHIN Information Document: 3.3 Tonga's experience in implementing national digital health. 2023. https://php.spc.int/sites/default/files/p-related-files/2023-03/2023%20PHIN%20Agenda%2013%20ID%203.3%20Tonga%20experience%20in%20implementing%20national%20digital%20health%20intervention%2028%20April%202023.pdf Date accessed: August 29, 2024.
89. World Health Organization. Advancing health information and digital transformation in the health sector (Fifteenth Pacific Health Ministers Meeting 20-22 September 2023) (draft). 2023. https://cdn.who.int/media/docs/default-source/wpro---documents/dps/phmm-phoh-documents/15th-phmm/advancing-health-information-and-digital-transformation-phmm-paper.pdf?sfvrsn=907f2b43_1 Date accessed: April 14, 2024.
90. World Health Organization. Pacific Health Information Network Regional Meeting on Strengthening Health Information Systems and Digital Health, Suva, Fiji, 28-30 March 2023. 2023. https://iris.who.int/bitstream/handle/10665/369972/RS-2023-GE-07-FJI-eng.pdf?sequence=1&isAllowed=y Date accessed: April 14, 2024.
91. Asian Development Bank. Tonga: Introducing eGovernment through Digital Health (50281-001). Project Administration Manual. March 2019. https://www.adb.org/sites/default/files/project-documents/50281/50281-001-pam-en.pdf Date accessed: August 24, 2024.
92. Asian Development Bank. Tonga: Introducing eGovernment through Digital Health (50281-001). Report and Recommendation of the President. July 2019. https://www.adb.org/sites/default/files/project-documents/50281/50281-001-rrp-en.pdf Date accessed: August 24, 2024.
93. Asian Development Bank. Tonga: Introducing eGovernment through Digital Health (50281-001). Concept Paper. March 2018. https://www.adb.org/sites/default/files/project-documents/50281/50281-001-cp-en.pdf Date accessed: August 24, 2024.
94. Pacific Community. Results Summary for Tonga 2022. https://spccfpstore1.blob.core.windows.net/digitallibrary-docs/files/2d/2dab048fc8ef6ce25f002135122678e1.pdf?sv=2015-12-11&sr=b&sig=GKypzEfYuI08YKGGtgHAy1VnvZsU4ujeSjATbF1Zd1M%3D&se=2025-05-31T04%3A47%3A59Z&sp=r&rscc=public%2C%20max-age%3D864000%2C%20max-stale%3D86400&rsct=application%2Fpdf&rscd=inline%3B%20filename%3D%22Tonga_Results_Summary_2022.pdf%22 Date accessed: August 29, 2024.
95. World Bank. Tuvalu Health System Strengthening Project (THSSP) Preliminary Environmental and Social Management Plan. 2022. https://mail.tuvaluwaste.tv/index.php/component/easyfolderlistingpro/?view=download&format=raw&data=eNpVUEEKwjAQ_ErZk55sK1TdnhUvgljxWmK7rYGYlCStgvh3E9NcIp6S

mdmdmYRhkuDLYIbQKFGThtzgBuFERvW6XCLjsRuB3pAOE4FCiFwnqe3J2N_2CqEsv5xH67Aq2Z08jBH8kQWW15BzjMOSJtExe_NymlwiLLZy4FrJO0lb_tVxlg0XNFmmrsL5Eu2L4hhti8Ox3HHJxCyZe3GJQE873rq6GQvSs-Pau4X0xNVi1rLq5tMgv35Z9w2dpoHTIzzMlW6VaoVLfX9cME_lYo0, Date accessed: May 18, 2024.
96. World Bank. Tuvalu Health System Strengthening Project Labour Management Procedure. 2022. https://finance.gov.tv/wp-content/uploads/2022/06/TV-HSSP-LMP_Final.pdf Date accessed: May 18, 2024.
97. World Bank. Concept Project Information Document (PID) - Health System Strengthening Project (Tuvalu) (P175170). Report No: PIDC32469. 2021. https://documents1.worldbank.org/curated/en/887751630457645388/pdf/Concept-Project-Information-Document-PID-Health-System-Strengthening-Project-P175170.pdf Date accessed: August 31, 2024.
98. World Bank. Project Information Document - Health System Strengthening Project (Tuvalu) (P175170). Report No: PIDA34123. 2022. https://documents1.worldbank.org/curated/en/099755005202235396/pdf/P1751700ffdc4b010a3450f482ce825550.pdf Date accessed: August 31, 2024.
99. World Bank. Implementation Status & Results Report - Health System Strengthening Project (Tuvalu) (P175170). Sequence No: 01. 2022. https://documents1.worldbank.org/curated/en/099035110192239467/pdf/P17517009106f50f5095110620f1bd2d8ab.pdf Date accessed: August 31, 2024.
100. World Bank. Implementation Status & Results Report - Health System Strengthening Project (Tuvalu) (P175170). Sequence No: 02. 2023. https://documents1.worldbank.org/curated/en/099051023182596064/pdf/P1751700c02da403d0aba10e4a110106036.pdf Date accessed: August 31, 2024.
101. World Bank. Implementation Status & Results Report - Health System Strengthening Project (Tuvalu) (P175170). Sequence No: 03. 2023. https://documents1.worldbank.org/curated/en/099101023093021774/pdf/P1751700c2ac050150940107f756e9a4a7e.pdf Date accessed: August 31, 2024.
102. World Bank. Implementation Status & Results Report - Health System Strengthening Project (Tuvalu) (P175170). Sequence No: 04. 2024. https://documents1.worldbank.org/curated/en/099041724223510336/pdf/P1751701d5f4730ff18ff2114488e4c1717.pdf Date accessed: August 31, 2024.
103. World Bank. Tuvalu Health Systems Strengthening Project: Project Appraisal Document. Report No: PAD5047. https://documents1.worldbank.org/curated/en/306951656370530051/pdf/Tuvalu-Health-Systems-Strengthening-Project.pdf Date accessed: August 31, 2024.
104. UNICEF. Mapping of Digital Health Tools and Technologies: Vanuatu Country Brief. 2021. https://drive.google.com/drive/folders/10znBRvotpfO5AMupJ09OVjJ_asqeYi4O Date accessed: August 17, 2024.
105. NTI and Brown University School fo Public Health Pandemic Center. Global Health Security Index Justification 2021: Vanuatu. 2021. https://ghsindex.org/wp-content/uploads/2021/12/Vanuatu.pdf Date accessed: August 18, 2024.
106. World Health Organization. Consultancy: Support for Development of Vanuatu HMIS (VanHMIS) (with focus on vaccination data management and AEFI surveillance) (advertisement). 2021. https://www.ungm.org/Public/Notice/126261 Date accessed: August 18, 2024.
107. World Health Organization. Consultancy: Strengthening DHIS2 Management Capacity Building in Vanuatu (advertisement). 2022. https://www.ungm.org/Public/Notice/186401 Date accessed: August 18, 2024.

108. Government of Vanuatu Ministry of Health. Health Information Strategic Plan 2016-2020. 2016. https://moh.gov.vu/images/health_policies/plans/Vanuatu_HIS_Strategic_Plan_2016_-_2020-_Final.pdf Date accessed: May 20, 2024.
109. Government of Vanuatu. Vanuatu Health Sector Strategy 2021-2030. https://moh.gov.vu/images/Strategic_Plan/Health_Sector_Strategy_HSS_2021-2030.pdf Date accessed: May 20, 2024.
110. Vanuatu Ministry of Health. Monitoring and Evaluating the HSS: Attachment to Vanuatu Health Sector Strategy (HSS), 2021-2030. https://moh.gov.vu/images/Strategic_Plan/Health_Sector_Strategy_HSS_2021-2030_ME_Plan.pdf Date accessed: May 18, 2024.
111. World Health Organization. SCORE for Health Data Technical Package: Vanuatu. 2021. https://cdn.who.int/media/docs/default-source/documents/ddi/score/country-profiles/who_score_vut_en.pdf?sfvrsn=ca7f3f56_13 Date accessed: May 4, 2024.
112. Asian Development Bank. Unlocking Innovation for Development. Action update: What worked and what didn't for ADB's first innovation regional technical assistance project. 2020. https://www.adb.org/sites/default/files/publication/568136/innovation-development-action-update.pdf Date accessed: April 21, 2024.
