# Supplementary material for: National Electronic Health Record Coverage in Pacific Island Countries and Territories: Environmental Scan
Source: J Med Internet Res. 2025 Oct 3;27:e71212. doi: 10.2196/71212 (PMC12534756; doi:10.2196/71212)
Supplement: Multimedia Appendix 5 [file jmir_v27i1e71212_app5.pdf]

Country	Economy income classification [GNI per capita, Atlas method]	National eHealth policy or strategy	National HIS policy or strategy	EHR system available	Year introduced	EHR coverage					
						Environmental scan results			Country validation results		
						Primary care facilities	*Secondary care facilities*	*Tertiary care facilities*	*National*	*Response to validation request*	*National EHR coverage [% difference]*
Solomon Islands	Lower-middle income[109] [2,270][110]	No	No	No	N/A	0% (0/283)	0% (0/9)	0% (0/1)	0% (0/293)	No	N/A
Papua New Guinea	Lower-middle income[109] [2,840][110]	Yes, not public (2017-2027) [4, 35-37]	No	Yes (Insta,[36] electronic Patient Medical Records Management System (ePMRMS),[36] Trias [personal correspondence, November 2024])	Unknown	0% (0/3198)	0% (0/14)	18.2% (4/22) [38]	0.1% (4/3234)	Yes	Results validated [0% difference]
Vanuatu	Lower-middle income[36] [3,660][110]	Outdated, not public (2019-2021) [39-42]	Outdated (2016-2020) [43]	No	N/A	0% (0/145)	0% (0/5)	0% (0/1)	0% (0/151)	Yes	Results validated [0% difference]
Kiribati	Lower-middle income[109] [3,730][110]	Outdated, not public (2018) [20, 44]	Outdated, not public (2012-2015) [4]	Yes (KHIS) [4, 44-47] Note: Tamanu implementation has recently begun[46]	2012 [4, 48]	0% (0/112)	33.3% (1/3) [44]	100% (1/1) [44, 45]	1.7% (2/116)	Yes	Results validated [0% difference]
Samoa	Lower-middle income[109] [4,020][110]	Outdated (2017-2022) [49]	Outdated (2017-2022) [49]	Yes (Tamanu) [50-58, 111]	2020 [53, 57, 59]	25% (1/4) [54, 60]	50% (3/6) [54, 60]	50% (1/2) [54, 60]	41.7% (5/12) [54, 60]	Yes	Results validated [0% difference]
Federated States of Micronesia	Lower-middle income[109] [4,150][110]	No	No	Yes (Unknown) [4, 61-68]	Unknown	0% (0/105)	0% (0/10)	100% (4/4) [62-66]	3.4% (4/119)	No	N/A
Tonga	Upper-middle income[109] [5,000][110]	No	No	Yes (Vesalius) [20, 21, 69]	2020 [21, 69]	29.2% (7/24) [20, 69]	25% (1/4) [20, 69]	100% (1/1) [20, 69]	31% (9/29)	Yes	78.1% (25/32) [151.8% increase]

Country	Economy income classification [GNI per capita, Atlas method]	National eHealth policy or strategy	National HIS policy or strategy	EHR system available	Year introduced	EHR coverage					
						Environmental scan results			Country validation results		
						Primary care facilities	*Secondary care facilities*	*Tertiary care facilities*	*National*	*Response to validation request*	*National EHR coverage [% difference]*
Fiji	Upper-middle income[109] [5,580][110]	Yes (2023-2027) [70]	Outdated (2016-2020) [71]	Yes (PATIS Plus) [4, 57, 70-77] *Tamanu is used at Lautoka and Ba Hospitals (operated by Aspen Medical under a public-private partnership with the Government of Fiji)[70]	2012 [70]	11.1% (21/190) [78]	83.3% (20/24)* [79]	100% (3/3)* [79]	20.3% (44/217)	No	N/A
Tuvalu	Upper-middle income[109] [7,550][110]	No	No	Yes (Hospital Management Information System [HMIS]) [80-87]	2013 [4]	0% (0/10)	N/A	100% (1/1) [80-87]	9.1% (1/11)	No	N/A
Marshall Islands	Upper-middle income[109] [7,570][110]	No	No	Yes (Marshall Hospital Information System [mHIS]) [88-91]	Unknown	0% (0/58)	N/A	50% (1/2) [88, 89]	1.7% (1/60)	No	N/A
Palau	High income[109] [14,250][110]	No	No	Yes (Tamanu) [92, 93, 111]	2022 [92]	0% (0/8)	N/A	100% (1/1) [92, 93]	11.1% (1/9)	Yes	Results validated [0% difference]
Niue	High income [16,478.1][94]	No	No	Yes (Medtech) [57, 95]	Unknown	N/A	100% (1/1) [57, 95]	N/A	100% (1/1)	Yes	Results validated [0% difference]
Cook Islands	High income [19,614.3][94]	No	Outdated (2015-2019) [96]	Yes (Medtech) [4, 21, 41, 57, 96-101]	2004 [41]	100% (15/15)	100% (1/1)	100% (1/1)	100% (17/17) [4, 96-98, 101]	No	N/A
Nauru	High income[109] [22,090][110]	No	No	Yes (Tamanu) [58, 102-105]	2021 [58, 102-104]	100% (3/3) [105]	N/A	100% (1/1) [58, 102-104]	100% (4/4)	No	N/A

Country	Economy income classification [GNI per capita, Atlas method]	National eHealth policy or strategy	National HIS policy or strategy	EHR system available	Year introduced	EHR coverage					
						Environmental scan results			Country validation results		
						Primary care facilities	*Secondary care facilities*	*Tertiary care facilities*	*National*	*Response to validation request*	*National EHR coverage [% difference]*
Total	Lower-middle: 6 Upper-middle: 4 High: 4	7.1% (1/14) in date and publicly available	0% (0/14) in date and publicly available	85.7% (12/14)	-	Results before validation					
						1.1% (47/4155)	35.1% (27/77)	46.3% (19/41)	2.2% (93/4273)	-	-
						Results after validation					
						1.5% (61/4158)	37.7% (29/77)	46.3% (19/41)	2.5% (108/4276)	50% (7/14)	[15.6% increase]

Coverage	Interpretation
0%	None
>0 to <25%	Low
≥25 to <50%	Medium
≥50 to <75%	High
≥75%	Very High
No facility type in country	N/A
